# A proof of concept infant-microbiota associated rat model for studying the role of gut microbiota and alleviation potential of *Cutibacterium avidum* in infant colic

**DOI:** 10.3389/fnut.2022.902159

**Published:** 2022-08-22

**Authors:** Vanesa Natalin Rocha Martin, Christophe Del’Homme, Christophe Chassard, Clarissa Schwab, Christian Braegger, Annick Bernalier-Donadille, Christophe Lacroix

**Affiliations:** ^1^Laboratory of Food Biotechnology, Department of Health Sciences and Technology, Institute of Food, Nutrition and Health, ETH-Zurich, Zurich, Switzerland; ^2^Division of Gastroenterology and Nutrition, University Children’s Hospital Zurich, Zurich, Switzerland; ^3^INRAE UMR 454, MEDIS Unit, Clermont-Ferrand Research Centre, Saint Genes-Champanelle, France; ^4^INRA UMR 545 Fromages, Aurillac, France

**Keywords:** infant gut microbiota, infant colic, human microbiota-associated rats, gnotobiotic, *Cutibacterium* (*Propionibacterium*) *avidum*, hydrogen

## Abstract

Establishing the relationship between gut microbiota and host health has become a main target of research in the last decade. Human gut microbiota-associated animal models represent one alternative to human research, allowing for intervention studies to investigate causality. Recent cohort and *in vitro* studies proposed an altered gut microbiota and lactate metabolism with excessive H_2_ production as the main causes of infant colic. To evaluate H_2_ production by infant gut microbiota and to test modulation of gut colonizer lactose- and lactate-utilizer non-H_2_-producer, *Cutibacterium avidum* P279, we established and validated a gnotobiotic model using young germ-free rats inoculated with fecal slurries from infants younger than 3 months. Here, we show that infant microbiota-associated (IMA) rats inoculated with fresh feces from healthy (*n* = 2) and colic infants (*n* = 2) and fed infant formula acquired and maintained similar quantitative and qualitative fecal microbiota composition compared to the individual donor’s profile. We observed that IMA rats excreted high levels of H_2_, which were linked to a high abundance of lactate-utilizer H_2_-producer *Veillonella*. Supplementation of *C. avidum* P279 to colic IMA rats reduced H_2_ levels compared to animals receiving a placebo. Taken together, we report high H_2_ production by infant gut microbiota, which might be a contributing factor for infant colic, and suggest the potential of *C. avidum* P279 in reducing the abdominal H_2_ production, bloating, and pain associated with excessive crying in colic infants.

## Introduction

Human cohort studies often reveal associations between the gut microbiota and host health, but valid *in vitro* and *in vivo* models are needed to provide mechanistic understanding (1). Animal models allow experimental procedures otherwise limited in humans due to ethical and safety concerns or due to limited accessibility to biological samples, while providing a controlled environment and standardized genetic background. Inoculation of germ-free animals with fecal microbiota from human donors has been used successfully to study the role of gut microbiota in various pathophysiological conditions. For example, Crouzet *et al.* (2) showed that the transfer of fecal microbiota from irritable bowel syndrome patients in germ-free rats was associated with increased abdominal sensitivity, compared to animals inoculated with fecal microbiota from healthy human subjects. Increased adiposity was also observed in germ-free mice that received fecal microbiota from obese individuals compared to mice inoculated with the microbiota of lean individuals (3). In a more recent study, germ-free mice inoculated with feces from patients suffering from inflammatory bowel disease showed a dysregulated immune response compared to mice receiving feces from healthy individuals (4). However, validated gnotobiotic models to study young infant gut microbiota are still lacking.

Infants are born with low numbers of microbes in their gut, and first colonizers are mainly transferred from the mother’s skin, mouth, vagina, and breast microbiota (5–7). The gut microbiota of breast- and formula-fed newborns is characterized by a high abundance of lactate-producing bacteria (LPB), mainly *Bifidobacterium, Streptococcus*, *Enterococcus*, and Enterobacteria (8–10). Lactate is an important intermediate metabolite that, if not further metabolized by lactate-utilizing bacteria (LUB), might accumulate leading to acidosis, neurotoxicity, and cardiac arrhythmia (11–13). The LUB community in infants is mainly composed of propionate-producing *Propionibacterium*/*Cutibacterium* and *Veillonella* spp., together with some species of the genus *Bacteroides* and with a lower abundance of butyrate-producing bacteria like *Anaerostipes* spp., *Anaerobutyricum hallii*, and *Eubacterium limosum*, and sulfate-reducing bacteria (SRB) like *Desulfovibrio* (8, 14–17). Hydrogen (H_2_), which is formed by *Anaerostipes* spp., *A. hallii*, and *Veillonella* spp., can accumulate and serve as a substrate for H_2_S formation by SRB (16, 15). Imbalances in lactate, H_2_, and H_2_S metabolism may induce flatulence and bloating and associated pain in colic infants (defined as infants younger than 5 months crying more than 3 h per day, for at least 3 days in a week) (16, 18–21). Several studies have identified higher breath H_2_ excretion in colic infants compared to healthy controls, and positively correlated breath H_2_ with crying time (22–24). Pham et al. (16) reported higher ratios of H_2_-producing to H_2_-utilizing LUB in crying infants compared to healthy controls at 3 months of age, and higher colonization by H_2_-producer *A. hallii* in colic infants compared to healthy controls at 2 weeks of age.

Because lactate is an important substrate for H_2_ production by LUB and accumulation of lactate and H_2_ might be contributing factors for infant colic (IC), supplementation of non-H_2_-producing LUB has been suggested as a potential strategy for IC alleviation. Pham et al. (16) demonstrated that infant isolates of non-H_2_-producing LUB *E. limosum* reduced H_2_ production by LUB *Veillonella ratii* when grown in co-culture in lactate-containing media. Alternatively, non-H_2_-producing LUB *Cutibacterium avidum* strains of infant origin have shown to persist, reduce lactate, and compete with *Veillonella* when supplemented in a recently developed and validated continuous colonic fermentation model (PolyFermS platform), inoculated with immobilized fecal microbiota and mimicking proximal colon conditions of 2-month-old formula-fed infants (17, 25). Considering the previous observations, we hypothesize that *Cutibacterium avidum* P279, selected for its colonization ability and effects observed in *in vitro* infant colonic fermentations, can decrease H_2_ excretion in a rat model of colic infants and has therefore potential for the treatment of IC. In order to test this hypothesis, we (1) developed and validated a rat model based on the inoculation of young germ-free animals with fecal slurries from healthy and colicky young infants (< 3 months) and (2) investigated the impact on gut microbiota composition and activity of supplementation of *C. avidum* P279 to colic infant microbiota-associated (IMA) rats.

## Materials and methods

### Donors and sample collection

**FIGURE 1:**
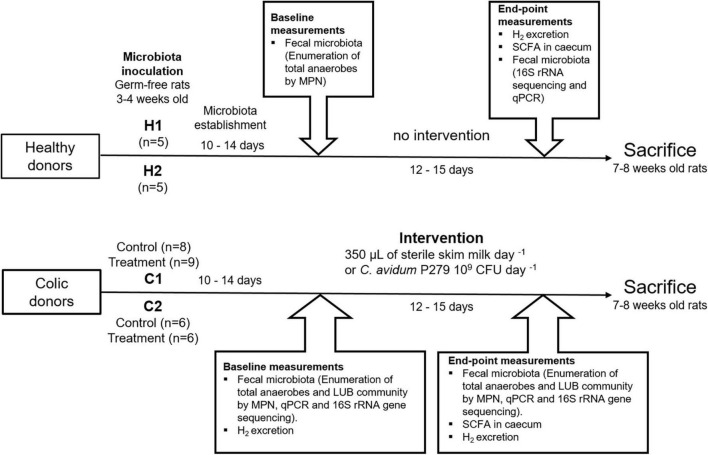
Diagram of the experimental design. Fecal samples from two healthy (H1 and H2) and two colic infants (C1 and C2) were used for inoculation of germ-free Fischer 344 rats (*n* = 39). The microbiota was allowed to establish for 10–14 days before baseline (T0) measurements. Rats inoculated with donors C1 and C2 were divided into treatment group (gavaged daily with 10^9^ colony forming units of *Cutibacterium avidum* P279 in 350 μL of sterile skim milk) and control group (received 350 μL of sterile skim milk). End-point measurements (T1) were obtained on days 26–28. Rats were sacrificed at 7–8 weeks of age. Rats were fed a gamma-irradiated infant formula to mimic an infant diet and supplemented with 1–3 pellets per day of standard diet for growing animals. All rats were given free access to sterile water.

Fecal samples from two healthy infants (H1: 59 days old and H2: 92 days old) and two infants suffering from colic (C1: 71 days old and C2: 68 days old) who satisfied Rome IV criteria (20) were used for inoculation of germ-free Fischer 344 rats (Figure 1). Inclusion criteria were as follows: good health, a full-term delivery (gestation time 37–42 weeks), normal birth weight (female: 2.7–5.0 kg; male: 2.9– 5.2 kg), and an exclusively milk-based diet. Infants with a history of antibiotic treatment were excluded. Healthy infants (H1 and H2) did not present gastrointestinal (GIT) disorders, while colic donors (C1 and C2) complied with the following criteria: i) caregiver reported infant crying or fussing, for more than 3 per day during 3 h or more days in the last week to the nurse; ii) total 24-h crying confirmed to be 3 or more h measured prospectively by caregivers; and iii) no evidence of failure to thrive (20). Infants were born by vaginal delivery and living in Zürich (Switzerland). Healthy donors were breastfed, while colic donors received infant formula complementary to breast-feeding. Parents of donors were asked to provide a fresh fecal sample under highly controlled conditions (presented below) to a study nurse, and the data and material were subsequently anonymized. Anonymized biological material is not under the scope of the Swiss Federal Human Research Act (Art. 2 para. 2 let. b and c), which excluded this study from requiring ethical approval. Fecal samples (2 - 4 g) were collected by the parents by scraping from diapers with a sterile spatula into 50-mL sterile conical-shaped polypropylene tubes and immediately placed into closed airtight jars (Mitsubishi AnaeroPack, Thermo Fisher Diagnostics AG, Pratteln, Switzerland) containing one AnaeroGen sachet (Oxoid, Thermo Fisher Diagnostic AG). Samples were kept under cold conditions until they were picked up from the family house and transported into a Styrofoam box with frozen gel packs to INRAE - Theix (France). Fecal slurries from healthy and colic donors were inoculated in germ-free rats after 24–36 h of fecal deposition.

### Bacterial strain, growth conditions, and preparation of supplementation doses

*Cutibacterium avidum* P279 was isolated from feces of a 13 week-old infant and selected for its colonization ability observed in *in vitro* infant colonic fermentations, and was obtained from the strain collection of the Laboratory of Food Biotechnology (ETH-Zürich) and used for supplementation of colic IMA rats (26). *C. avidum* P279 was grown in yeast extract sodium lactate medium (YEL) consisting of 1% (w/v) trypticase soy broth without dextrose (Becton Dickinson AG, Allschwil, Switzerland), 1% (w/v) yeast extract (Merck, Darmstadt, Germany), 117 mM sodium DL-lactate (60% v/v syrup, Central Drug House, New Delhi, India), 0.025% (w/v) KH_2_PO_4_ (VWR International AG, Dietikon, Switzerland), and 0.0005% (w/v) MnSO_4_ (Sigma-Aldrich, Buchs, Switzerland). Glycerol stocks stored at -80°C were reactivated on agar plates and incubated in airtight jars (Mitsubishi AnaeroPack, Thermo Fisher Diagnostics) containing one AnaeroGen sachet (Oxoid, Thermo Fisher Diagnostics AG) at 37°C for 5 days. Single colonies were inoculated into liquid YEL and incubated for 48 h at 37°C. After incubation, cultures were centrifuged for 10 min at 7,000 g, and pellets were resuspended in the same volume of sterile reconstituted skim milk (10% w/v). Aliquots of 350 μL (containing approximately 10^9^ CFU) were transferred into cryovials (Huber Co, Reinach, Switzerland). For supplementation of the control group, aliquots of 350 μL of sterile reconstituted skim milk (10% w/v) were prepared in cryovials (Huber Co). Cryovials were snap-frozen in liquid nitrogen and stored at -80°C for a maximum of 5 weeks until supplementation.

### Animal experiments

Germ-free Fischer 344 rats bred at INRAE-Theix facilities were kept in sterile isolators with positive pressure over the entire trial period as described before (2). All experimental protocols were approved by the Local Institutional Animal Care and Use Committee (Approval reference N° 2019101512192520).

Rats were weaned at 3–4 weeks of age and inoculated with 0.2 mL of infant fecal slurries from single donors by a single intragastric gavage [100-fold dilution of fecal sample in anaerobic mineral solution (27) that was composed of 7.5% v/v mineral solution I (0.3% w/v K_2_HPO_4_), 7.5% v/v mineral solution II [0.3% w/v K_2_HPO_4_; 0.6% w/v NaCl; 0.6% w/v (NH_4_)_2_SO_4_; 0.06% w/v MgSO_4_; 0.06% w/v CaCl_2_], 0.01% w/v resazurin, 0.4% w/v NaHCO_3_, 0.05% w/v cysteine hydrochloride, and distilled water; all chemicals were obtained from Sigma-Aldrich]. IMA rats were placed in cages housing two animals. They were fed with gamma-irradiated infant formula (Aptamil Pre, Milupa GmbH, Germany) reconstituted in sterile water (13% w/v; approximately 80 mL per day during the first 2 weeks and afterwards 100 mL per day until the end of experimentation) mimicking infant diet. The commercial formula contained 8% (w/v) galacto-oligosaccharides (GOS) and fructo-oligosaccharides (FOS) (ratio of 9:1). Two to three pellets of the standard diet for growing rats (A03/R03, SAFE, France) per day were also supplied to each cage (after 2 weeks from weaning for healthy IMA rats and during entire intervention period for colic IMA rats) in order to prevent bloating due to the lack of indigestible fibers and high content of lactose of the infant formula. All rats were given free access to sterile water.

Ten germ-free rats were inoculated with fecal slurries from healthy donors H1 (*n* = 5) and H2 (*n* = 5), and were allocated to two different isolators (Figure 1). Seventeen rats inoculated with fecal slurries from colic donor C1 were divided into two isolators housing a control group (*n* = 8) and a treatment group (*n* = 9). Fecal slurries from colic donor C2 were used to inoculate 12 rats housed in two isolators (control group *n* = 6 and treatment group *n* = 6).

After inoculation, the microbiota was established for a period of 10–14 days before further measurements (Figure 1).

### Sample collection from infant microbiota-associated rats

Fecal samples from rats were collected directly from the anus by manual perineal stimulation and were immediately used for microbial enumeration (see below) or stored at –80°C until further processing. Feces collection and fecal microbial enumeration were done 10–14 days from inoculation (baseline measurements) and at the end of experimentation (Figure 1). After baseline measurements, rats inoculated with fecal microbiota from donors C1 and C2 were gavaged daily for 12–15 days with 10^9^ CFU of *C. avidum* P279 in 350 μL of sterile skim milk (treatment groups) or with the same amount of sterile milk alone (control groups). Rats were sacrificed at 7-8 weeks of age, and the cecum contents were harvested for analyzing fermentation metabolites.

### Enumeration of total bacteria and functional bacterial groups

Freshly voided feces from donors and rats were serially 10-fold diluted (wet w/v) in an anaerobic mineral solution. Total anaerobes, and non-SRB-LUB and SRB-LUB communities were enumerated by most probable number estimation (MPN) in a three-replicate design (28). Total anaerobes in all donors and rat feces were enumerated in an O_2_-free CO_2_ complex medium containing clarified rumen fluid (29). Oxygen-free CO_2_ basal medium with L-lactate (35 mM) as the sole energy source and Postgate E medium were used for the enumeration of non-SRB-LUB and SRB-LUB communities, respectively (30, 31). After incubation for 5 days at 37°C, lactate concentration in supernatant of culture tubes of each inoculated dilution (10^–5^ to 10^–10^) was determined by high-performance liquid chromatography with refractive index detection (HPLC-RI) (described below). Cultures with a final lactate concentration below 25 mM (lactate consumption of at least 10 mM) were considered positive for non-SRB-LUB bacteria (32). Cultures on Postgate E medium were inoculated with 0.3 mL of fecal dilutions (10^–2^ to 10^–5^) and were considered positive for MPN determination when a black precipitate of FeS was visually observed.

### DNA extraction and quantitative PCR analysis

Genomic DNA was extracted from infant and rat stool samples (200 mg) and from MPN-positive cultures (2 mL) in Postgate E and basal medium with L-lactate, using the Fast DNA SPIN kit for soil (MP Biomedicals, Illkirch, France) according to the manufacturer’s instructions. The extracted DNA was then used to amplify regions of the 16S rDNA gene by qPCR using specific primers (Supplementary Table 1). Reactions were performed using LightCycler 480 Real-Time PCR System (Roche Diagnostics, Rotkreuz, Switzerland) and the SensiFAST SYBR No-ROX 2X mix (5 μL), and 500 nM primers (Biolab Scientifics Instruments SA, Chatel-St-Denis, Switzerland) in a total reaction volume of 10 μL. Thermal cycling started with an initial denaturation step at 95°C for 3 min, followed by 40 cycles of a two-step PCR consisting of a hold at 95°C for 5 s and at 60°C for 60 s. The cycle threshold (Ct) values were obtained using automatic baseline and threshold settings provided by the LightCycler 480 Software, Version 1.5. Samples were analyzed in duplicate. To generate standards, PCR amplicons were cloned into the pGEM-T Easy Vector and heterologously expressed in *E. coli* according to instructions of the supplier (Promega AG, Dübendorf, Switzerland). Standard curves were prepared from 10-fold dilutions of linearized plasmids harboring the target gene of interest. Bacterial groups abundant in the infant feces were amplified using primers listed in Supplementary Table 1. Melting curve analysis was conducted to confirm amplification specificity. To estimate cell counts for *Cutibacterium*, gene copies were corrected for multiple copies (*n* = 3) of 16S rRNA genes (33).

### 16S rRNA gene amplicon sequencing

The bacterial profiles in fecal samples obtained from donors and rats and in pellets from cultures in Postgate E and basal medium with lactate were determined using tag-encoded 16S rRNA gene Miseq-based (Illumina, San Diego, CA, United States) high-throughput sequencing, as presented before (17). Briefly, DNA concentration was standardized to 20 ng μL^–1^. The V3 region of the 16S rRNA gene was amplified using primers including adapters for the Nextera Index Kit, NXt_388_F, and NXt_518_R (Supplementary Table 1). One MiSeq flow cell and the V2 2 × 250 bp paired-end Nextera chemistry were used, and were supplemented with 20% of PhiX. Library preparation and sequencing were performed at the Genomic Diversity Center (ETH Zurich, Zurich, Switzerland). The raw datasets containing pair-ended reads with corresponding quality scores were merged and trimmed using settings as previously described (34). The minimum length of merged reads was 200 bp. Following analysis steps were done using Quantitative Insight Into Microbial Ecology (QIIME) open source software package (1.8.0 and 1.9.0) (35). Purging the datasets from chimeric reads and constructing *de novo* operational taxonomic units (OTUs) were conducted using the UPARSE pipeline (36) and the Greengenes database as a reference (37). Taxonomic assignment of OTUs at the genus level could not differentiate between *Propionibacterium* and *Cutibacterium* because the new taxonomy (a division of former *Propionibacterium* spp. in gen. nov. *Cutibacterium* including cutaneous species and *Propionibacterium* spp. including only dairy species) has not been updated in the reference database (38). Alpha-diversity was analyzed using observed species (species richness in individual samples) and Shannon index (species richness and evenness estimator, which increase when the number of species and evenness increase). The beta-diversity (compositional diversity between two samples) was analyzed as previously described using iterative subsampling (39). Permutational multivariate analysis of variance using distance matrices (PERMANOVA) based on 999 Monte Carlo simulations was used to analyze the differences in unweighted and weighted UNIFRAC distances between infant and IMA rat fecal samples from different donors. Linear discriminant analysis effect size (LEfSe) was applied to identify genera that differed significantly between IMA rats inoculated with fecal samples from the same donor, from treatment and control groups at baseline (T0) and after the intervention period (T1). LEfSe analysis was done with default parameters and a logarithmic linear discriminant analysis score threshold of two (40).

### Metabolite analysis by high-pressure liquid chromatography-refractive index detection

The concentration of acetate, propionate, butyrate, isobutyrate, isovalerate, formate, succinate, and lactate were determined in cecum content and in the supernatant of LUB enumeration media after centrifugation for 10 min at 13,000 g. The supernatant (500 μL) was filtered through a 0.45-μm membrane (Millipore AG, Zug, Switzerland) into glass HPLC vials (Infochroma, Hitachi LaChrome, Merck, Dietikon, Switzerland) and sealed with crimp caps. An HPLC (Hitachi LaChrome) equipped with a Security Guard Cartridges Carbo-H column (4 × 3 mm; Phenomenex Inc., Torrance, CA, United States), a Rezex ROA-Organic Acid H + column (8%, 300 × 7.8 mm; Phenomenex), and a refractive index detector (HPLC-RI) was used. The column was eluted with 10 mM H_2_SO_4_ (Fluka, Buchs, Switzerland) as a mobile phase at a flow rate of 0.4 mL min^–1^ at 25°C. All tested metabolites were quantified using external standards using EZChrom Elite software (version 3.3.2 SP2, Agilent Technologies, CA, United States).

### Rats hydrogen excretion

The amount of H_2_ excreted by each rat was measured by housing a single animal in a respiratory chamber for 24 h (41). Measurements were done on days 27 and 28 for rats inoculated with feces from healthy donors (H1 and H2) and before (baseline measurements T0, 10–14 days from inoculation) and after treatment (end-point measurements T1, after 12-15 days of intervention) for rats inoculated with feces from colic donors (C1 and C2). H_2_ concentration in the chamber atmosphere was determined using a gas phase chromatograph (Microanalyzer DP, Quintron Instruments, Milwaukee, WI, United States).

### Statistical analysis

Statistical analyses were done using SigmaPlot (Systat Software, San Jose, CA, United States) with statistical significance set at a *p*-value of less than 0.05. The Student’s *t*-test with two-tailed distribution was used, after testing for normal distribution using the Shapiro–Wilk test, to compare changes in the abundance of microbial taxa detected by qPCR (log_10_-transformed), cecum metabolites (mmol L^–1^) and H_2_ excretion (ppm) values between baseline and after intervention for the same IMA rat group and between control and treatment IMA rat groups after the intervention period. The Mann–Whitney test was used when data were not normally distributed.

### Availability of supporting data

Sequence information is available in the NCBI database, BioProject ID PRJNA590392.

## Results

### Fecal microbiota of infant donors

**FIGURE 2:**
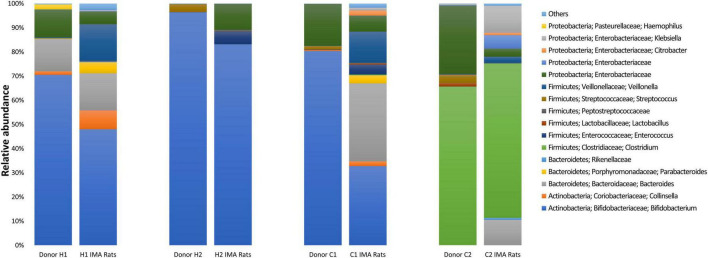
Bacterial community composition was determined by 16S rRNA gene amplicon sequencing in feces from infant donors and respective infant microbiota-associated (IMA) rats. For IMA rats, each column represents the average relative abundance. The relative abundance of taxa with an average relative abundance of >0.1% are depicted with the taxa nomenclature; lower abundant taxa (< 0.1%) are shown as Others.

The bacterial composition in feces of infant donors was analyzed using qPCR and 16S rRNA gene amplicon sequencing. Species richness in donors ranged from 10 to 46 observed species (Table 1). *Bifidobacterium* [Log 9.6 gene copies g feces^–1^, 70.5% relative abundance (rel. ab.)] and *Bacteroides* (Log 10.2 gene copies g feces^–1^, 13.4% rel. ab.) were the most abundant genera in donor H1, while *Bifidobacterium* dominated in donor H2 (Log 10.1 gene copies g feces^–1^, 96.4% rel. ab.) and C1 (Log 8.9 gene copies g feces^–1^, 80.3% rel. ab.) (Figure 2 and Table 1). Donor C2 fecal microbiota was dominated by Firmicutes (Log 9.1 gene copies g feces^–1^, 70.7% rel. ab.), mainly represented by *Clostridium* cluster I (Log 9.0 gene copies g feces^–1^). *Enterobacteriaceae* were detected in high abundance in donors H1, C1, and C2 (Log 8.0-8.8 gene copies g feces^–1^ and 11.6 - 29.1% rel. ab.) but at low concentrations in donor H2 (Log 3.8 gene copies g feces ^–1^, < 0.0001% rel. ab.). *Veillonella* was identified in all donors at sub-dominant levels (Log 4.6-7.5 gene copies g feces ^–1^, 0.01 - 0.3% rel. ab.) (Figure 2 and Table 1). The donor fecal samples used to inoculate IMA rats are representative of the highly variable taxonomic composition of the infant gut microbiota.

**TABLE 1:** Main bacterial communities in feces from infant donors and IMA rats at baseline and fermentative metabolites in cecal supernatant from IMA rats after euthanasia.

	Healthy microbiota				Colic microbiota			
	H1		H2		C1		C2	
	Donor	IMA rats (*n* = 5)	Donor	IMA rats (*n* = 5)	Donor	IMA rats (*n* = 10)	Donor	IMA rats (*n* = 10)
**Cultivable bacteria (Log cells g feces^–1^)**								
Total anaerobes	11.5	11.0 (0.1)*^A^*	10.2	10.5 (0.1)*^A^*	9.5	10.6 (0.5)	9.6	10.4 (0.5)
non-SRB LUB	ND	ND	ND	ND	ND	10.1 (0.3)	ND	10.4 (0.3)
SRB LUB	ND	ND	ND	ND	BDL	BDL	4.4	BDL
**Fecal microbiota α -diversity (16S rRNA gene amplicon sequencing)**								
observed species	46	51 (3)	10	17 (1)	32	56 (2)	24	26 (3)
Shannon Index	3.3	3.8 (0.4)	0.8	2.0 (0.2)	3.2	4.4 (0.2)	2.5	2.8 (0.3)
**Abundance of bacterial taxa (Log gene copies g feces^–1^)**								
Total bacteria	10.5	11.0 (0.1)	10.2	10.7 (0.2)	9.0	10.0 (0.1)	8.9	9.3 (0.2)
Firmicutes	8.6	9.8 (0.1)	8.6	9.5 (0.2)	9.0	9.6 (0.2)	9.1	9.6 (0.2)
*Enterobacteriaceae*	8.8	9.0 (0.4)	3.8	9.0 (0.2)	8.0	8.5 (0.6)	8.2	8.1 (0.4)
*Clostridium* cluster I	BDL	BDL	BDL	BDL	BDL	BDL	9.0	10.4 (0.2)
*Bacteroides* spp.	10.2	10.7 (0.1)	5.2	5.1 (0.3)	8.2	10.4 (0.1)	7.1	9.4 (0.4)
*Bifidobacterium*	9.6	10.4 (0.2)	10.1	10.3 (0.2)	8.9	10.0 (0.3)	6.5	5.9 (0.4)
*Veillonella*	6.1	9.8 (0.2)	5.0	3.8 (0.4)	4.6	9.7 (0.2)	7.5	8.6 (0.5)
**Microbial metabolites in rat caecum**								
Acetate	ND	73.2% (2.4%)	ND	67.3% (13.8%)	ND	72.5% (4.2%)*^B^*	ND	43.2% (5.4%)*^B^*
Propionate	ND	15.8% (6.1%)	ND	2.1% (4.7%)	ND	21.2% (5.0%)	ND	23.1% (5.7%)
Butyrate	ND	2.0% (4.1%)	ND	5.3% (1.6%)	ND	0.6% (0.5%)	ND	33.7% (6.8%)
Formate	ND	4.6% (5.5%)	ND	1.3% (2.9%)	ND	2.8% (1.8%)	ND	BDL
Lactate	ND	4.4% (1.5%)	ND	24.0% (14.4%)	ND	3.0% (5.5%)	ND	BDL

Data are shown as mean ± SD. ^*A*^ Enumerated in two rats from each group.^*B*^ Values detected in rats from control group. Quantification by qPCR in donor feces was done on the same sample used for inoculation of IMA rats. ND, not determined. Metabolites in donor samples could not be determined due to small sample amount. BDL, below detection limit; LUB, lactate-utilizing bacteria.

### Colonization of infant fecal microbiota in germ-free rats

Viable cell numbers in donor slurries used for inoculating germ-free rats were estimated by an MPN-based cultivation approach. Slurries from healthy donors had higher viable cell numbers at inoculation (H1: Log 11.5 cells g feces^–1^ and H2: Log 10.2 cells g feces^–1^) than colic donors (C1: Log 9.5 cells g feces^–1^ and C2: Log 9.6 cells g feces^–1^). After implantation, viable cell numbers of healthy and colic IMA rats were very similar, averaging Log 10.6 ± 0.5 cells g feces^–1^, while some differences were observed among donor feces using MPN (Table 1).

Quantification of bacterial groups in IMA rats by qPCR revealed higher levels of Firmicutes (Log 9.5–9.8 gene copies g feces^–1^) in rats compared to donors (Table 1). The levels of other bacterial taxa were donor-dependent and generally matched the composition of the donor microbiota. *Bifidobacterium* colonized H1, H2, and C1 rats at high abundance (Log 10.0–10.4 gene copies g feces^–1^) and C2 rats at a much lower level (Log 5.9 ± 0.4 gene copies g feces^–1^) similar to donor C2 (Log 6.5 gene copies g feces^–1^). Engraftment of *Bacteroides* in H1 rats (Log 10.7 ± 0.1 gene copies g feces^–1^) and H2 rats (Log 5.1 ± 0.3 gene copies g feces^–1^) also corresponded with donor fecal concentrations (H1: Log 10.2 gene copies g feces^–1^ and H2: Log 5.2 gene copies g feces^–1^). *Bacteroides* were enriched approximately 100-fold in C1 (Log 10.4 ± 0.1 gene copies g feces^–1^) and C2 rats (Log 9.4 ± 0.4 gene copies g feces^–1^) compared to respective donors (C1: Log 8.2 gene copies g feces^–1^ and C2: Log 7.1 gene copies g feces^–1^). *Enterobacteriaceae* were well maintained in H1, C1, and C2 rats, but reached a much higher number in H2 rats (Log 9.0 ± 0.2 gene copies g feces^–1^) than in donor H2 which had a very low number of *Enterobacteriaceae* (Log 3.8 gene copies g feces^–1^) compared to the other donors. *Veillonella* was much higher in H1 (Log 9.8 ± 0.2 gene copies g feces^–1^), C1 (Log 9.7 ± 0.2 gene copies g feces^–1^), and C2 (Log 8.6 ± 0.5 gene copies g feces^–1^) rats compared to the respective donors (H1: Log 6.1 gene copies g feces^–1^, C1: Log 4.6 gene copies g feces^–1^, and C2: Log 7.5 gene copies g feces^–1^) but not in H2 rats (Log 3.8 ± 0.4 gene copies g feces^–1^) compared to donor H2 (Log 5 gene copies g feces^–1^) (Table 1).

The number of observed species and Shannon indices were higher in rats compared to donors (Table 1). Except for C1 rats, fecal microbiota communities from IMA rats and respective donors were qualitatively similar, as indicated by the clustering of donor samples together with recipient rats when considering unweighted and weighted UniFrac distances in a principal component analysis (PCoA) plot (Supplementary Figure 1). When comparing groups of donors and respective recipient rats, cluster separation by donors was significant under unweighted and weighted UniFrac distances (PERMANOVA, *p* = 0.001) (Supplementary Figure 1).

Main shifts in the bacterial populations identified by gene amplicon sequencing were aligned with qPCR observations (Figure 2). *Bacteroidaceae* increased from 0.02% in donor C1 to 32.2% ± 14.8% in C1 rats and from 0.07% in donor C2 to 10.5% ± 8.4% in C2 rats. Colonization by *Enterobacteriaceae* was higher in feces of H2 rats (10.7% ± 3.5%) compared to donor H2 (< 0.01%) (Figure 2). Higher abundance of *Veillonellaceae* was also detected for H1 (15.4% ± 5.0%), C1 (12.9% ± 5.9%), and C2 rats (2.4% ± 1.6%) compared to respective donor levels (H1: 0.3%, C1: 0.1%, and C2: 0.2%) (Figure 2). In contrast, shifts in the relative abundance of *Bifidobacteriaceae* detected by sequencing were not aligned with qPCR observations for donors H1 and C1 and the corresponding IMA rats. In contrast to qPCR, the members of *Bifidobacteriaceae* were at a lower relative abundance in H1 rats (48.0% ± 11.4%) than in donor H1 (70.5%) and C1 rats (32.7% ± 9.1%) compared to donor C1 rats (80.3%) (Figure 2).

Overall, dominant microbes and microbiota profiles of infant donors were transferred and established with similar composition in H1, H2, and C2 rats as shown by qPCR and sequencing data. Microbiota engraftment in C1 rats measured with qPCR was consistent with donor microbiota composition, but differences among C1 rats were observed with sequencing, with an overall increased abundance of *Bacteroidaceae* compared to donor C1 feces.

### Microbial fermentation activity in infant microbiota-associated rats

Different metabolic profiles were detected in cecal water from rats inoculated with different donors by HPLC-RI. Acetate (73.2% ± 2.4%) and propionate (15.8% ± 6.1%) were main metabolites identified in H1 rats, while acetate (67.3% ± 13.8%) and lactate (24.0% ± 14.4%) were the main metabolites detected in H2 rats. In colic rats, acetate (72.5% ± 4.2%) and propionate (21.2% ± 5.0%) were the main SCFAs identified in the cecal supernatant of C1 rats, while acetate (43.2% ± 5.4%), butyrate (33.7% ± 6.8%), and propionate (23.1% ± 5.7%) were the main SCFAs in C2 rats (Table 1).

**FIGURE 3:**
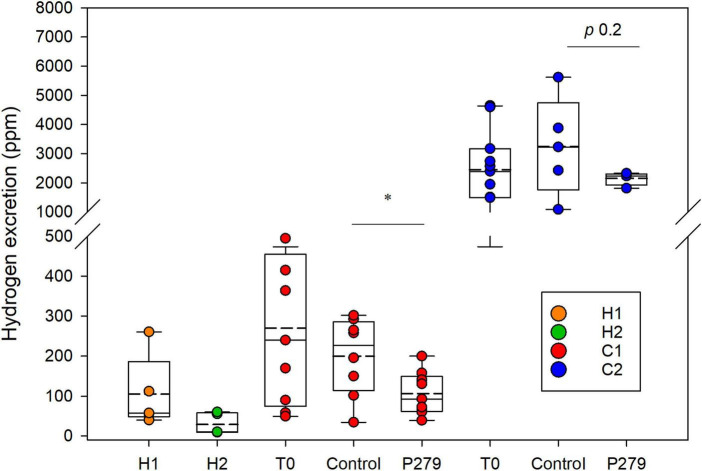
Hydrogen excretion by IMA rats. Hydrogen concentration (ppm) accumulated in the respiratory chambers after 24 h of housing single IMA rats. Measurements were done at the end of experimentation for rats inoculated with feces from healthy donors (H1 and H2) and at baseline (T0) and after intervention for rats inoculated with feces from colic donors (C1 and C2). Treatment groups (P279) were gavaged daily for 12–15 days with 10^9^ CFU of *Cutibacterium avidum* P279 in 350 μL of sterile skim milk, and control groups received daily 350 μL of sterile skim milk. Values are mean (H1 *n* = 5; H2 *n* = 5; C1 T0 *n* = 9; C1 P279 *n* = 9; C1 control *n* = 8; C2 T0 *n* = 11; C2 P279 *n* = 4; and C2 control *n* = 5). Central horizontal solid lines show the median and dashed lines the mean, upper and lower box borders show the 90th and 10th centiles, respectively, and upper and lower whiskers show the 95th and 5th centiles, respectively.

After 2 weeks of inoculation, H_2_ accumulation was measured in respiratory chambers after housing single IMA rats for a 24-h period. H1 rats excreted higher H_2_ levels (105.2 ± 91.3 ppm) compared to H2 rats (28.6 ± 26.4 ppm; *p* 0.09) (Figure 3). However, significantly higher amounts of H_2_ were measured in colic than in healthy IMA rats (C1 rats control group: 200 ± 97.3 ppm and C2 rats control group: 3,248.0 ± 1,686.9 ppm; *p* 0.001) (Figure 3). Rats inoculated with feces from donor C2 excreted significantly higher levels of H_2_ than the C1 rats (*p* 0.002).

### Lactate-utilizing bacteria abundance in colic infant microbiota-associated rats

The LUB community was enumerated in colic IMA rat feces at baseline using an MPN-based cultivation approach (Table 1). Non-SRB LUB colonized C1 (Log 10.1 ± 0.3 cells g feces^–1^) and C2 rats (Log 10.4 ± 0.3 cells g feces^–1^) at comparable and high levels. 16S rRNA gene amplicon sequencing of pellets from MPN cultures (in basal medium with L-lactate) detected mainly *Veillonella* (82.5 ± 5.6% rel. ab.) followed by *Enterobacteriaceae* (8.9 ± 3.9%) and *Bacteroides* (2.0 ± 1.8%). Acetate and propionate were the main products of lactate metabolism, which was in agreement with the bacterial populations recovered.

The SRB LUB (Log 4.4 cells g feces^–1^) cultured in Postgate E medium from feces of C2 donor were mainly identified as *Desulfovibrio* (80.1 ± 0.0% rel. ab.). SRB LUB were below detection limit in C2 rat feces at all time points and were not detected in feces from donor C1 and corresponding C1 rats.

### Impact of *Cutibacterium avidum* P279 on fecal microbiota composition and metabolic activity of colic infant microbiota-associated rats

The effect of daily supplementation of 10^9^ CFU *C. avidum* P279 in sterile milk, a non-H_2_-producing LUB, after 12–15 days of intervention on gut microbiota composition and metabolism was tested in colic IMA rats and compared to control groups receiving only sterile milk. The colonization of *C. avidum* P279 in IMA rats from treatment groups was tested by qPCR and 16S rRNA gene amplicon sequencing. Donor-dependent effects were observed on microbiota composition and metabolism.

*Cutibacterium avidum* P279 colonized C1 rats with fecal abundance of Log 7.5 ± 0.2 cells g feces ^–1^ (Table 2). LEfSe analysis based on 16S rRNA gene amplicon sequencing of the fecal microbiota of C1 rats also identified an increase in *Propionibacteriaceae* in the supplemented rats after the intervention period compared to baseline (Supplementary Figure 2D). When treated C1 rats were compared to the control group, LEfSe analysis identified increased *Propionibacteriaceae* in the supplemented rats, while *Veillonellaceae* abundance was higher in the control rats (Supplementary Figure 2B). In both groups, the *Enterobacteriaceae* population decreased after the intervention period, which corresponded with a significant reduction of Log 1.0 gene copies g feces^–1^ (Table 2 and Supplementary Figures 2C,D). Interestingly, formate and H_2_ excretion were significantly lower in the treatment group compared to control animals (0.4 ± 0.6 mM vs. 1.7 ± 1.2 mM for formate, respectively, *p* 0.02; 106.3 ± 53.8 ppm vs. 200.0 ± 97.3 ppm for H_2_ production, respectively, *p* 0.01) (Figure 3 and Table 2).

**TABLE 2:** Main bacterial communities in feces from colic IMA rats at baseline and after intervention.

	C1 IMA rats				C2 IMA rats			
	Control (*n* = 8)		Treatment (*n* = 9)		Control (*n* = 8)		Treatment (*n* = 9)	
	Baseline	End-point	Baseline	End-point	Baseline	End-point	Baseline	End-point
**Cultivable bacteria (Log cells g feces^–1^)**								
Total anaerobes	10.7 (0.5)	11.2 (0.1)*	10.4 (0.4)	10.5 (0.3)*	10.3 (0.6)	11.0 (0.3)*^†^	10.5 (0.4)	10.5 (0.3)*
non-SRB LUB	10.2 (0.3)*^A^*	10.2 (0.5)	9.9 (0.1)*^A^*	10.2 (0.6)	10.3 (0.2)	10.5 (0.0)^†^	10.5 (0.3)	10.1 (0.7)
SRB LUB	BDL	BDL	BDL	BDL	BDL	BDL	BDL	BDL
**Abundance of bacterial taxa (Log 16S rRNA gene copies g feces^–1^)**								
Total bacteria	10.0 (0.2)	9.6 (0.2)*^†^	10.0 (0.1)	9.8 (0.1)*^†^	9.3 (0.1)	9.3 (0.1)	9.4 (0.2)	9.2 (0.1)
Firmicutes	9.6 (0.3)	9.2 (0.2)*^†^	9.6 (0.2)	9.5 (0.2)*	9.5 (0.2)	9.3 (0.2)	9.7 (0.2)	9.2 (0.2)^†^
*Clostridium* cluster I	BDL	BDL	BDL	BDL	10.3 (0.2)	10.0 (0.1)	10.5 (0.2)	10.0 (0.2)^†^
*Enterobacteriaceae*	8.3 (0.3)	7.3 (0.5)^†^	8.6 (0.7)	7.6 (0.6)^†^	8.2 (0.4)	8.1 (0.2)	7.9 (0.3)	8.1 (0.3)
*Bacteroides* spp.	10.5 (0.2)	10.2 (0.2)^†^	10.3 (0.1)	10.3 (0.2)	9.4 (0.4)	9.5 (0.1)	9.3 (0.4)	9.4 (0.2)
*Bifidobacterium*	9.9 (0.4)	9.9 (0.3)	10.0 (0.3)	10.0 (0.2)	6.0 (0.4)	5.7 (0.4)	5.8 (0.5)	5.7 (0.2)
*Veillonella*	9.6 (0.2)	9.8 (0.1)^†^	9.7 (0.2)	9.7 (0.2)	8.6 (0.5)	8.9 (0.2)	8.7 (0.3)	8.6 (0.5)
*Cutibacterium*	BDL	BDL	BDL	7.5 (0.2)	BDL	BDL	BDL	5.9 (0.3)
**Microbial metabolites in rat caecum**								
Acetate	ND	72.5% (4.2%)	ND	77.8% (4.9%)	ND	43.2% (5.4%)	ND	47.1% (5.6%)*
Propionate	ND	21.2% (5.0%)	ND	19.4% (4.3%)	ND	23.1% (5.7%)	ND	27.9% (8.4%)*
Butyrate	ND	0.6% (0.5%)	ND	0.5% (0.7%)	ND	33.7% (6.8%)	ND	22.3% (8.0%)
Formate	ND	2.8% (1.8%)	ND	0.5% (1.0%)*	ND	BDL	ND	1.7% (2.6%)
Lactate	ND	3.0% (5.5%)	ND	1.8% (5.1%)	ND	BDL	ND	1.0% (1.6%)

Data are shown as mean ± SD. Treatment groups were gavaged daily for 12–15 days with 10^9^ CFU of C. avidum P279, and control groups received 350 μL of sterile skim milk. ^A^ Enumerated in two rats from each group. LUB: lactate-utilizing bacteria. ND: not determined. BDL: below detection limit. ^†^p < 0.05: comparison between fecal samples at baseline and after intervention within the same IMA rat group. *p < 0.05: comparison between fecal samples from control and treatment IMA rat groups from the same donor at the same intervention period.

*Cutibacterium avidum* P279 colonized C2 rats with fecal abundance of Log 5.4 ± 0.3 cells g feces^–1^, which was about log 2 lower than in C1 rats (Table 2). After treatment, the abundance of Firmicutes and *Clostridium* cluster I was significantly reduced by Log 0.5 ± 0.2 and Log 0.5 ± 0.2 gene copies g feces^–1^ (*p* < 0.01), respectively (Table 2). In agreement, LEfSe analysis identified a decrease in *Clostridiaceae*, and an increase of *Propionibacteriaceae* in treated animals. In C2 rats, *Veillonellaceae* increased after *C. avidum* P279 supplementation (Supplementary Figure 3D). The abundance of *Propionibacteriaceae* and *Veillonellaceae* was higher in the C2 treated than in the control rats, while the abundance of *Bifidobacteriaceae* and *Enterobacteriaceae* was lower (Supplementary Figure 3B).

Acetate and propionate levels, but not butyrate, were significantly higher in the C2 treatment group (11.9 ± 2.9 mM, 7.2 ± 3.3 mM, and 5.6 ± 2.2, respectively) compared to the control animals (acetate 6.4 ± 2.5 mM, *p* < 0.01; propionate 3.3 ± 0.8 mM, *p* < 0.05; and butyrate 5.1 ± 2.7 mM *p* > 0.05) after intervention. We measured a high although not significant reduction of the mean H_2_ production in the treated C2 rats with *C. avidum* P279 compared to the rats from the control group, which is likely due to the large spread observed in the control group (2,152.5 ± 228.96 ppm vs. 3,248.0 ± 1,686.86 ppm, respectively; *p* 0.2). Surprisingly, much lower variability in H_2_ excretion among the treated animals (1,815–2,325 ppm) was observed compared to the control rats (1,085–5,620 ppm) (Figure 3).

## Discussion

Human microbiota-associated animal models are valuable tools to study the effect of the resident gut microbiota on host pathobiology, and to perform invasive procedures that are not possible in humans due to ethical constraints. In this study, we focused on the establishment of a rat model for studying gut microbiota in infants younger than 3 months and tested the modulation potential of *C. avidum* P279 supplementation on H_2_ excretion in colic IMA rats. In general, microbiota diversity and taxonomic composition in IMA rats and donors were similar, while inter-individual differences in the microbiota composition could be reproduced.

### Infant microbiota establishment in germ-free rats

We inoculated 3- to 4- week-old germ-free rats with fresh fecal microbiota collected from healthy and colic donors ensuring high viability at inoculation by storing fresh samples immediately after deposition under anaerobic conditions and at cooling temperatures. We chose to use feces from single donors and not pooled samples for inoculation, as there is a high inter-individual variability of the gut microbiota during the first year of life (8, 14, 28). The gut microbiota of the four donors participating in this study was representative of infant profiles dominated by *Bifidobacteriaceae* and *Clostridiaceae* as reported previously (14, 28, 42). The donors gut microbiota profiles were overall highly conserved in IMA rats. The fecal microbiota profiles of C1 rats identified by 16S rRNA gene amplicon sequencing were discordant with qPCR findings for *Bifidobacterium* and *Bacteroides*. The bias can be partly explained by the occurrence of species with different numbers of 16S rRNA gene copies within the targeted taxa, and differences in the efficiency of primers to amplify different members within a taxonomic group (43). The metabolic profiles identified in the ceca of rats corresponded with the metabolic capacity of the most abundant fecal colonizers identified. Preserving donor’s individuality in models used to investigate gut microbiota modulation strategies is of high relevance, as the effect of such interventions might be dependent on the individual microbiome characteristics as it has been previously shown in *in vitro* fermentation models (25). This is the first report of IMA rat model. The model will be helpful for studying the gut microbiome of young infants with a personalized approach.

### Diet and host physiology influence infant microbiota colonization in IMA rats

The individual microbiota profiles of donors, H1, H2, and C2, were preserved in IMA rats, while C1 rats showed decreased *Bifidobacteriaceae* and enriched *Bacteroides*. *Bifidobacteriaceae* and *Bacteroides* are common infant colonizers that are able to degrade HMOs (10, 44–46). Providing IMA rats with infant formula that lacked HMOs and contained FOS and GOS could be a contributing factor to the quantitative differences observed in the establishment of these taxa in C1 recipient rats. Furthermore, important differences in the morphology and physiology of the rat gastrointestinal system compared to infants, as well as individual and variable digestive and environmental conditions in infants, can also explain differences between infant donors and IMA rat fecal microbiota. A lower relative abundance of *Bifidobacterium* and higher colonization of *Bacteroides* were recently reported for IMA mice and piglets inoculated with fecal samples from a 5-month-old infant (47). The differential establishment of these two core infant taxa might be also explained by differences in the host physiology that could determine bacterial colonization ability.

Working with rats instead of mice has a clear advantage in our goal to model microbiota in young animals (3–4 weeks old). The small size of mice at that same age would not have allowed to handle and gavage the animals and obtain reliable measurements of fecal samples and respiratory activities. Moreover, rats develop slower than mice, which was an advantage for our study which involved testing a 2-week supplementation in a model of infant colic. Rats reach puberty at day 50 after birth (48), while mice reach it at 28 days (49).

We initially fed IMA rats inoculated with the microbiota of healthy donors with only infant formula, but severe bloating was observed for some animals after approximately 2 weeks, possibly due to a surplus of rapidly metabolized di- and oligosaccharides (lactose, FOS, and GOS) and a lack of fibers in the infant formula. The appearance of bloating was coincidental with previous reports of decreased lactase expression and activity in rats from day 21 of life (50). Supplementation with a standard chow diet (two to three pellets per cage per day) from the second week of experimentation improved this condition, probably due to a bulking effect of the dietary fibers included in the pellets as reported before, although some animals still showed little cecal content and bloating at euthanasia (51).

The high availability of lactose, FOS, and GOS might have supported LPB metabolism resulting in high lactate concentrations, which would explain the higher abundance of *Veillonella*, an H_2_-producing LUB identified in H1, C1, and C2 rats compared to the donor levels. Strikingly, *Veillonella* remained low in rats inoculated with feces from H2 donor, suggesting limited enrichment when originally present at low abundance.

### Metabolite profiles in infant microbiota-associated rats were reflective of dominant bacterial populations

Acetate and propionate or lactate are the main fermentation metabolites identified in feces of infants younger than 6 months of age (28, 52, 53). In agreement, acetate was the main microbial metabolite identified in the ceca of all rats, while propionate was also produced in the ceca of H1 and C1 rats, which could be linked to a high abundance of propionate-producing *Bacteroides* and *Veillonella* (54). Lactate was the other metabolite identified with acetate in the ceca of H2 rats in alignment with the dominant colonizer LPB *Bifidobacterium*, while butyrate detection in C2 rats might be due to the high presence of butyrate producer *C. neonatale* (64% rel. ab.) belonging to *Clostridium* cluster I, as identified in C2 donors and rats by 16S rRNA gene amplicon sequencing.

### H_2_ production in infant microbiota-associated rats was high and could be linked to H_2_-producing species

The H_2_ production varied greatly according to the IMA rat group. H_2_ excretion of H2 rats was the lowest in agreement with the lowest abundance of lactate-utilizer H_2_-producer *Veillonella*. The extremely high H_2_ production in C2 rats might be due to *C. neonatale* which is able to ferment lactose to acetate, lactate, and butyrate while also producing H_2_. *In vitro* gas production by *C. neonatale* infant isolates during milk fermentation has been reported (55).

Surprisingly, compared to previous experiments in germ-free rats inoculated with fecal microbiota from healthy adults and irritable bowel syndrome patients (12.3 ± 7.3 ppm and 33.6 ± 3.6 ppm excreted H_2_, respectively) (2), H_2_ released by IMA rats was up to 100-fold higher. High breath H_2_ excretion values were reported for ca. 30% of non-colic and ca. 60% of colic infants due to physiologic lactose malabsorption followed by gut microbial metabolism (22–24, 56). The higher H_2_ excretion detected for colic IMA rats compared to healthy IMA rats could result from a functional dysbiosis in relation to high production and accumulation of H_2_, as has been proposed for colic infants previously (16, 22–24). More trials should be done with different donors, both healthy and colicky, to corroborate these findings.

### Lactose- and lactate-utilizer non-H_2_-producing *Cutibacterium avidum* P279 as candidate probiotic for infant colic treatment

Colic infants are diagnosed based on their crying time (more than 3 h per day, during at least 3 days in a week). Studies consider an intervention to be successful if crying time is reduced by at least 50% compared to the baseline values (20, 57, 58). The number of trials reporting the supplementation of probiotics in IC has steadily increased in recent years, but until now there is no clear evidence of beneficial effects of any strains/products on crying time reduction (20, 59, 60).

In this study, we evaluated infant gut colonizer *C. avidum* P279 as a candidate probiotic for inhibition of H_2_ production for IC treatment, based on its capability to metabolize lactose and lactate to propionate and acetate without producing H_2_, and on the hypothesis that imbalances in lactate and H_2_ metabolism may be the cause of bloating and associated pain in colic infants (16). We observed a reduction and lower variability in H_2_ excreted by colic IMA rats supplemented with *C. avidum* compared to control animals receiving the sterile milk carrier.

In an already propionigenic gut microbiota (C1), the SCFA profile in rat feces indicated a slightly lower formation of formate, but a significant reduction in excreted H_2_ and *Veillonellaceae* abundance in colic C1 rats compared to the control animals. We have recently reported that the addition of *C. avidum* P279 to an *in vitro* colonic fermentation also led to a lower abundance of *Veillonella* and formate production (17).

On the other hand, in C2 IMA rats, *C. avidum* P279 supplementation shifted the SCFA profile from a butyrogenic to higher propionate concentrations with an observed high decrease in H_2_ excretion, although not significant due to the high variability observed in the control rats. Interestingly, lower variability in H_2_ excretion was also observed in treated compared to non-treated C2 rats. Supplementation with *C. avidum* P279 in C2 IMA rats decreased colonization of dominant *Clostridium* in treated rats after the intervention compared to baseline values. These observations might have been consequence of a possible competition for lactose between *C. avidum* P279 and H_2_-producing *Cl. neonatale* (26, 55). *Cl. neonatale* has only recently been isolated and described as main colonizer in preterm infants (9, 55). *C. avidum* P279 also increased the abundance of taxa involved in lactate metabolism in C2 IMA rats, mainly LPB *Lactobacillaceae* and LUB *Veillonellaceae*. We previously reported the modulation in the lactate trophic chain by a *C. avidum* strain of infant origin, in an *in vitro* PolyFermS model mimicking infant proximal colon conditions and inoculated with fecal microbiota from a 2-month-old infant, with enhanced lactate consumption and increased LUB *Eubacterium limosum* (25).

Overall, our results indicate that *C. avidum* P279 supplementation can reduce H_2_ excretion and donor-dependently modulate the gut microbiota composition and metabolic activity in colic IMA rats, with no adverse effects observed on the health status of IMA rats.

## Conclusion

We showed for the first time that fecal microbiota composition and functional profiles of single infants younger than 3 months old could be transferred upon transplantation to young germ-free rats fed infant formula and supplemented with a conventional chow diet. Our results indicate that this gnotobiotic rat model is suitable for investigating the role of gut microbiota in the nutrition and health of young infants. By transferring fecal microbiota of milk-fed infants to germ-free rats, we first report an increased level of H_2_ production by infant microbiota *in vivo*, compared to adult microbiota, and a very high H_2_ excretion in colic IMA rats. Furthermore, a pronounced reduction in H_2_ excretion was shown after supplementing *C. avidum* P279, lactose- and lactate-utilizing non-H_2_-producing strain, supporting the potential of the strain to reduce abdominal H_2_ production and IC-associated symptoms. Further experiments using this rat model with additional infant donors could help to identify the range of effects that the strain could have on different microbiota profiles. In order to confirm the potential efficacy of *C. avidum* P279 in colic alleviation, crying time reduction has to be evaluated in human clinical trials previous safety assessment of the strain.

## Data availability statement

The datasets presented in this study can be found in online repositories. The names of the repository/repositories and accession number(s) can be found in the article/Supplementary material.

## Ethics statement

The animal study was reviewed and approved by Local Institutional Animal Care and Use Committee.

## Author contributions

VRM, CD, CC, AB-D, and CL designed the experiments. VRM and CD performed the gnotobiotic experiments and data analysis. VRM, CS, AB-D, and CL supported with result interpretation. VRM wrote the manuscript which was subsequently edited and revised by all authors. All authors contributed to the article and approved the submitted version.
