# Supplementary material for: A proof of concept infant-microbiota associated rat model for studying the role of gut microbiota and alleviation potential of Cutibacterium avidum in infant colic
Source: Front Nutr. 2022 Aug 22;9:902159. doi: 10.3389/fnut.2022.902159 (PMC9441890; doi:10.3389/fnut.2022.902159)
Supplement: Supplementary file 1 [file Data_Sheet_1.docx]

Supplementary Material

## Supplementary Figures

**Supplementary Figure S1.** Alpha-diversity and beta-diversity identified by 16S rRNA gene amplicon sequencing in infant donors and respective IMA rats fecal samples at the end of the implantation period. Donor H1 (orange), H2 (green), C1 (red) and C2 (blue). A) Mean observed species (count of unique OTUs in each sample) detected in IMA rats and respective donors. B) Mean Shannon Index (richness and evenness estimator, the index increases when the number of species increases and when evenness increases) identified in IMA rats and respective donors. C) Principal coordinate analysis based on unweighted and weighted (D) UniFrac distances between donors (large symbols) and respective IMA rat fecal samples (small symbols). Permutational multivariate analysis of variance (PERMANOVA) using weighted and unweighted distance matrices based on 999 Monte Carlo simulations indicated significant (p 0.001) compositional differences between samples from infants and IMA rats from the same donor compared to samples from different donors.

Supplementary Figure S2. Linear discriminant analysis effect size (LEfSe) of differentially abundant taxa in fecal samples of C1 IMA rats from control and treatment groups at baseline (T0) and after intervention (T1). Cladograms (on the left) represent statistically and biologically consistent taxonomical differences between compared groups. Circle’s diameter is proportional to the taxon’s abundance. Histograms (on the right) depict the logarithm (base 10) linear discriminant axis (LDA) scores computed for differentially abundant taxa between compared groups. LDA scores absolute values can be interpreted as the degree of consistent difference in relative abundance between the two groups. Differences of the most abundant taxon are represented in the respective color of each group.

Supplementary Figure S3. Linear discriminant analysis effect size (LEfSe) of differentially abundant taxa in fecal samples of C2 IMA rats from control and treatment groups at baseline (T0) and after intervention (T1). Cladograms (on the left) represent statistically and biologically consistent taxonomical differences between compared groups. Circle’s diameter is proportional to the taxon’s abundance. Histograms (on the right) depict the logarithm (base 10) linear discriminant axis (LDA) scores computed for differentially abundant taxa between compared groups. LDA scores absolute values can be interpreted as the degree of consistent difference in relative abundance between the two groups. Differences of the most abundant taxon are represented in the respective color of each compared group.


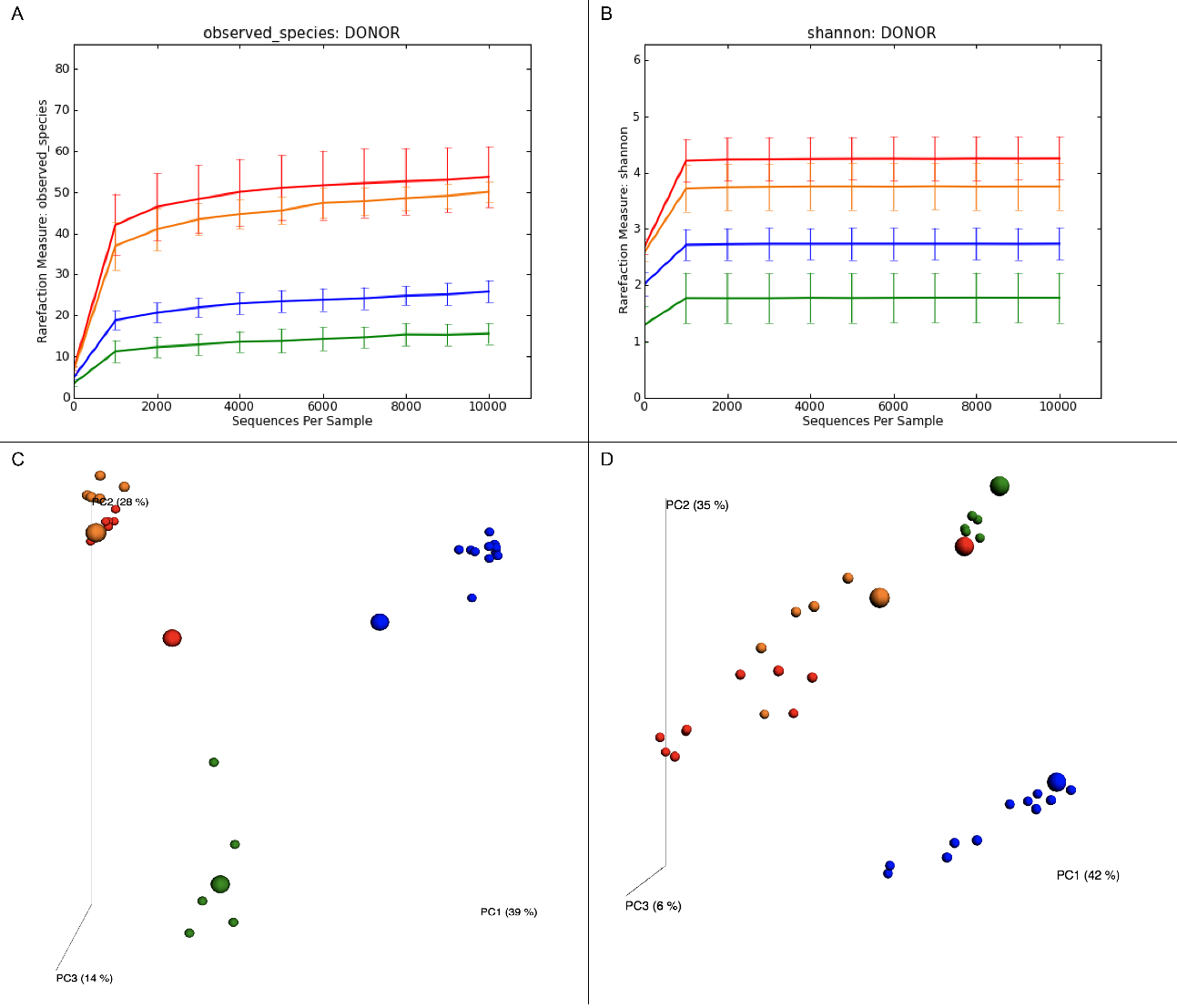
**Supplementary Figure S1.** Alpha-diversity and beta-diversity identified by 16S rRNA gene amplicon sequencing in infant donors and respective IMA rats fecal samples at the end of the implantation period. Donor H1 (orange), H2 (green), C1 (red) and C2 (blue). A) Mean observed species (count of unique OTUs in each sample) detected in IMA rats and respective donors. B) Mean Shannon Index (richness and evenness estimator, the index increases when the number of species increases and when evenness increases) identified in IMA rats and respective donors. C) Principal coordinate analysis based on unweighted and weighted (D) UniFrac distances between donors (large symbols) and respective IMA rat fecal samples (small symbols). Permutational multivariate analysis of variance (PERMANOVA) using weighted and unweighted distance matrices based on 999 Monte Carlo simulations indicated significant (p 0.001) compositional differences between samples from infants and IMA rats from the same donor compared to samples from different donors.


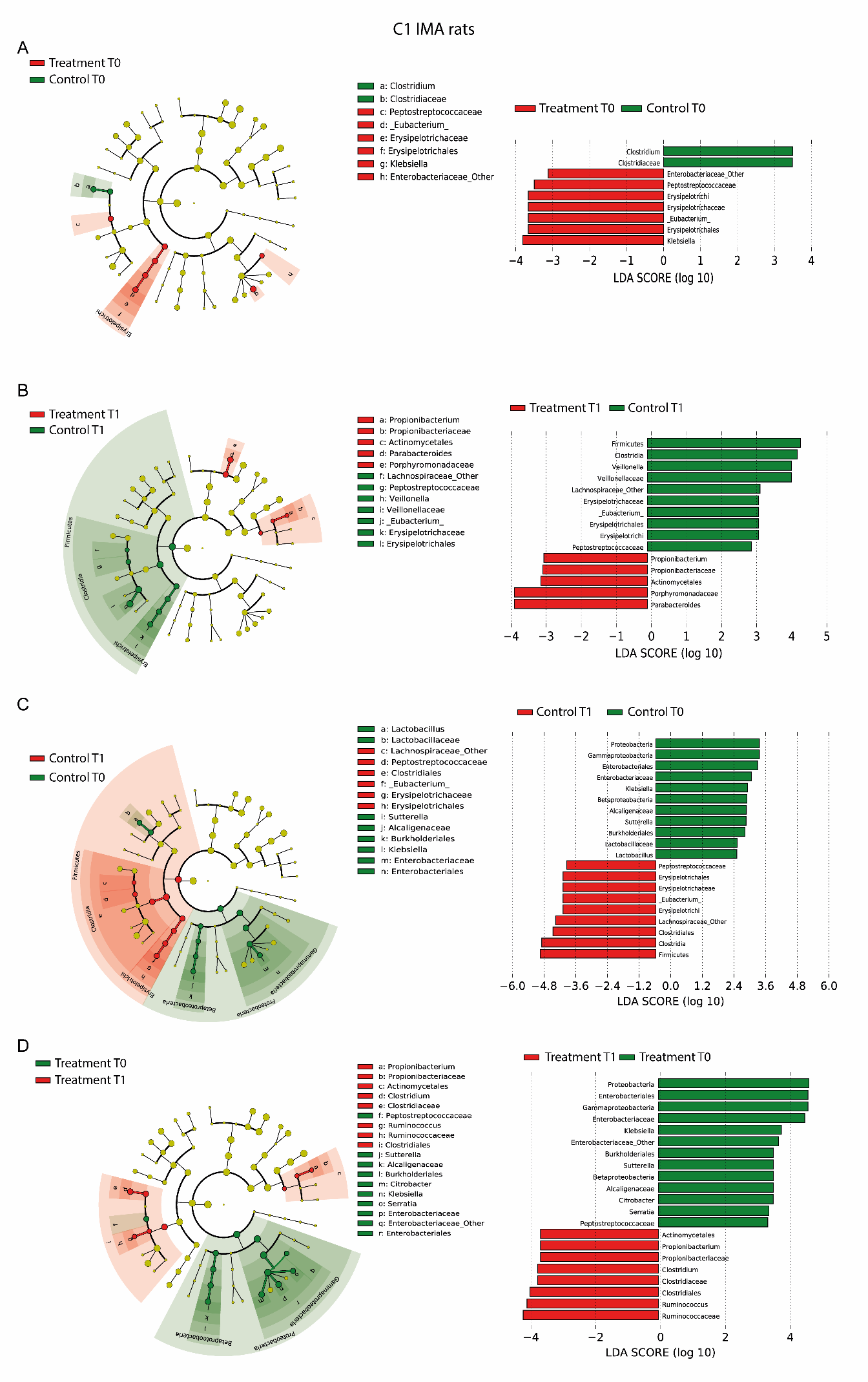


Supplementary Figure S2. Linear discriminant analysis effect size (LEfSe) of differentially abundant taxa in fecal samples of C1 IMA rats from control and treatment groups at baseline (T0) and after intervention (T1).


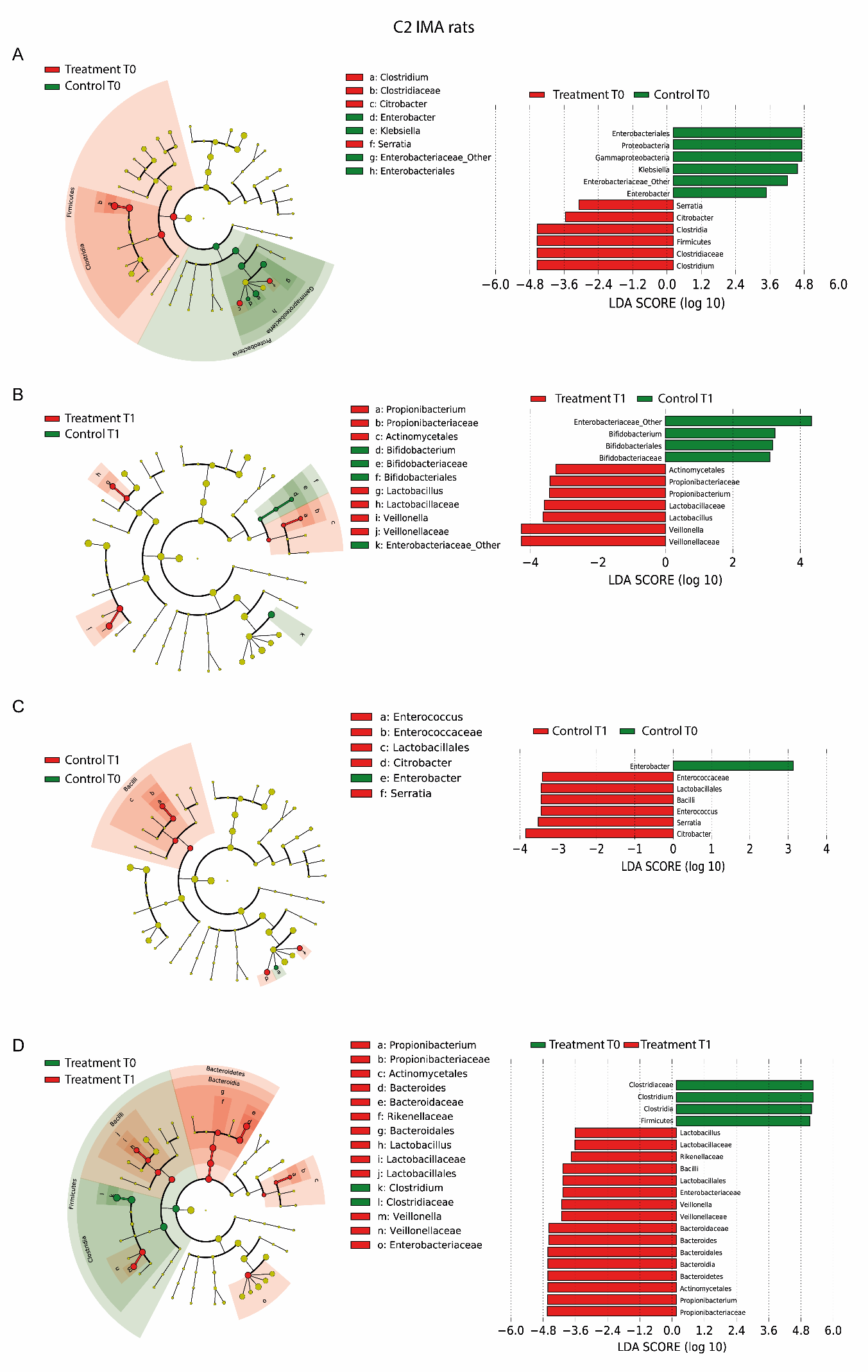


**Supplementary** **Figure S3. Linear discriminant analysis effect size (LEfSe) of differentially abundant taxa in fecal samples of C2 IMA rats from control and treatment groups at baseline (T0) and after intervention (T1).**

**Supplementary Table S1. List of primers used in this study.**

| **Target organism** | **Primers** | **5'-3'** | **Standard** | **Reference** |
| --- | --- | --- | --- | --- |
| *Veillonella* spp. | Vspp - F | AYCAACCTGCCCTTCAGA | *V. rattii* | Price et al., 2007 |
|  | Vspp - R | CGTCCCGATTAACAGAGCTT |  |  |
| *Bacteroides* spp | Bac303F | GAA GGT CCC CCA CAT TG | *B. thetaiotaomicron* | Ramirez-Farias et al., 2009 |
|  | Bfr-Femrev | CGC KAC TTG GCT GGT TCAG |  |  |
| *Enterobacteriaceae* | Eco1457F | CAT TGA CGT TAC CCG CAG AAG AAG C | *E. coli* | Bartosch et al., 2004 |
|  | Eco1652R | CTC TAC GAG ACT CAA GCT TGC |  |  |
| *Bifidobacterium* | Bif F | TCGCGT CYGGTGTGAAAG | *B. adolescentis* | Rinttilä et al., 2004 |
|  | Bif R | CCACATCCAGCRTCCAC |  |  |
| *Firmicutes* | Firm934F | GGA GYA TGT GGT TTA ATT CGA AGC A | *R. intestinalis* | Guo et al., 2008 |
|  | Firm1060R | AGC TGA CGA CAA CCA TGC AC |  |  |
| Total Bacteria | Eub338F | ACTCCTACGGGAGGCAGCAG | *R. intestinalis* | Fierer et al., 2005 |
|  | Eub518R | ATTACCGCGGCTGCTGG |  |  |
| *Propionibacterium* and *Cutibacterium* | Avi Fwd | GTCTGCAACTCGACCCCAT | *C. avidum* | Rocha Martin et al., 2019a |
|  | Avi Probe | CTTCGACGGCTCCCCCACACAGGT |  |  |
| *Clostridium* cluster I | fwd | ATG CAA GTC GAG CGA KG | *Cl. prefringens* | Rinttilä et al., 2004 |
|  | rev | TAT GCG GTA TTA ATC TYC CTT |  |  |
| 16S rRNA gene - V3 region | NXt_388_F | TCGTCGGCAGCGTCAGATGTGTATAAGAGACAGACWCCTACGGGWGGCAGCAG | | Illumina Miseq technology (Illumina, San Diego, CA, USA) |
|  | NXt_518_R | GTCTCGTGGGCTCGGAGATGTGTATAAGAGACAGATTACCGCGGCTGCTGG | |  |
| All oligonucleotides used in this study were obtained from Microsynth (Balgach, Switzerland). | | | | |
